# Surface electromyography pattern of human swallowing

**DOI:** 10.1186/1472-6831-8-6

**Published:** 2008-03-26

**Authors:** Annalisa Monaco, Ruggero Cattaneo, Alessandro Spadaro, Mario Giannoni

**Affiliations:** 1 Department of Gnathology and Prostethic Dentistry,_School of Dentistry, University of L'Aquila-Italy; 2 Department of Odontostomatological Clinic, School of Dentistry, University of L'Aquila-Italy

## Abstract

**Background:**

The physiology of swallowing is characterized by a complex and coordinated activation of many stomatognathic, pharyngeal, and laryngeal muscles. Kinetics and electromyographic studies have widely investigated the pharyngeal and laryngeal pattern of deglutition in order to point out the differences between normal and dysphagic people. In the dental field, muscular activation during swallowing is believed to be the cause of malocclusion.

Despite the clinical importance given to spontaneous swallowing, few physiologic works have studied stomatognathic muscular activation and mandibular movement during spontaneous saliva swallowing.

The aim of our study was to investigate the activity patterns of the mandibular elevator muscles (masseter and anterior temporalis muscles), the submental muscles, and the neck muscles (sternocleidomastoid muscles) in healthy people during spontaneous swallowing of saliva and to relate the muscular activities to mandibular movement.

**Methods:**

The spontaneous swallowing of saliva of 111 healthy individuals was analyzed using surface electromyography (SEMG) and a computerized kinesiography of mandibular movement.

**Results:**

Fifty-seven of 111 patients swallowed without occlusal contact (SNOC) and 54 individuals had occlusal contact (SOC). The sternocleidomastoid muscles showed a slight, but constant activation during swallowing. The SEMG of the submental and sternocleidomastoid muscles showed no differences between the two groups. The SEMG of the anterior temporalis and masseter muscles showed significant differences (p < 0.0001). The duration of swallowing was significantly higher in the SNOC subjects. Gender and age were not related to electromyographic activation. Healthy SOC and SNOC behaved in different ways.

**Conclusion:**

The data suggest that there is not a single "normal" or "typical" pattern for spontaneous saliva swallowing. The polygraph seemed a valuable, simple, non-invasive and reliable tool to study the physiology of swallowing.

## Background

The balanced muscular activity in the development of the face has been recognized for many years.

Many authors have stated that the tongue and its function, swallowing, is very important in obtaining muscular and skeletal equilibrium. The muscular forces could directly mold cranial and facial development and could affect teeth position [1,2].

The mandibular anchorage to the cranium is a primary physiologic event that allows the action of the tongue and of the suprahyoid muscles on the hyoid bone during all phases of swallowing. The hyoid bone moves upward during the first phase of swallowing; at the same time, the mandible moves upward too to reach occlusal contact. At the end of deglutition, the hyoid bone moves downward and the mandible, leaving occlusal contact, also moves downward and reaches its rest position [3,4]. Mandibular stabilization on the cranium requires an isometric contraction of the mandibular elevator muscles. This work could be obtained both with the occlusal contact and with the tongue interposition between the dental arches.

Moreover, during swallowing, the neck muscles help to stabilize the cranium on the thorax, counterbalancing the propulsive forces exerted by the tongue, pharynx, and larynx [5].

In dental research and in orthodontics it has been accepted for long time that saliva swallowing could be divided into two groups: Typical and Atypical swallowing. Deglutition without occlusal contact is one of the characteristics of Atypical swallowing. "Atypical" swallowing has been considered one of the malocclusion causes and its correction was suggested by some Clinicians in order to solve the "malocclusion". The idea that only "Typical" deglutition would be a correct deglutition induced to believed that "Atypical" deglutition is pathologic and need some kind of treatment. If malocclusion is a disease, its cause (Atypical deglutition) has to consider a pathological problem too. The "Typical" and the "Atypical" swallowing concept is based on spontaneous saliva swallowing occurring during the night and the day. These definitions, currently, didn't concern the swallowing of solid or liquid boluses occurring during feeding. Swallowing difficulties during feeding could be considered a particular case in dental practice. "Atypical" swallowing, in this case, it is a restrictive definition used in dental field.

Surface electromyography (SEMG) has been widely used in recent years to study the physiology of swallowing. Recent studies have suggested that the SEMG graphic record is a valid and reliable support in identifying normal swallowing [6]. The rectified and filtered SEMG represents a non-invasive tool to assess certain aspects of the complex muscle activity involved in deglutition. The SEMG for swallowing is a simple and reliable, non-invasive screening method for evaluating swallowing with low levels of discomfort [7,8].

The electrodes placed on the anterior belly of the digastric muscle record the electrical activity from both the anterior digastric muscles and the suprahyoid area muscles (i.e., the geniohyoid and mylohyoid muscles). All these muscles fire during swallowing [9]. Swallowing is a frequent physiologic act. Vaiman stated that the rate of spontaneous swallowing of saliva in healthy individuals is once every 2 minutes and 15 seconds [10]. The strength, duration, and frequency of contraction of the tongue muscles during swallowing are related to the morphology of the bones and teeth of the stomatognathic system, like the open bite or other kinds of malocclusion [2]. EMG activation precedes the swallowing biomechanical events and it is strictly connected to these. The strongest relationship is between the elevation and the anterior displacement of the hyoid bone and the SEMG signal of the submental muscles [11]. The duration of swallowing ranges from 0.80 to 1.60 seconds, according to the measurement techniques [3,12]. The same temporal pattern is shown in the SEMG of the submental muscles. This normal duration is achieved by the age of 12 years and does not change significantly until 70 years of age. After this age, the duration of swallowing shows a significant increase [7,11,13,14]. Ertekin et al. [15] suggested that spontaneous saliva swallowing differs from voluntarily dry swallowing both in neurological control and in sub mental muscles activation.

During isometric work, the SEMG values are related in a non-linear way to the force and to the work generated by the muscles [16,17]. This situation is similar to mandibular stabilization during swallowing.

Many SEMG works concerning both the stomatognathic and the neck muscular activity during clenching or mastication in healthy or temporomandibular disorders (TMD) patients exist in the literature [18-21]. Relatively few works, however, have studied the SEMG behaviour of the stomatognathic and neck muscles during swallowing [22,23].

Furthermore, these works have not simultaneously considered SEMG activity and mandibular movement and could not relate isometric activity of the stomatognathic and neck muscles with an objective analysis of the occlusal contact or of the tongue interposition between the dental arches during swallowing.

In recent years, computerized kinesiography has allowed objective recordings of mandibular movement during swallowing [19,24,25].

At present, with polygraph equipment, it is possible to record mandibular movement and SEMG activity at the same time.

The aim of our study was to investigate the mandibular movement and the activity pattern of the mandibular elevator muscles (i.e., masseter and anterior temporalis muscles) and the submental and neck muscles (i.e., sternocleidomastoid muscles) in healthy individuals during the spontaneous swallowing of saliva. A further aim of the study was to investigate the relative frequency and polygraph differences of spontaneous saliva swallowing pattern, separating people who swallows with polygraph occlusal contact (SOC) from those who swallows without occlusal contact (SNOC).

The null hypothesis, is that the mean values of the rectified SEMG and the mean time of swallowing would be statistically the same.

## Methods

One hundred eleven patients (71 females and 40 males), with an average age of 32.7 years (sd, 14.1), registered for an annual dental check-up at the Dentistry Department of the University of L'Aquila, were selected for the study. A clinical dental evaluation assured the presence of all dental elements. Complete or partial denture-wearers and patients with wide reconstructions or fixed prostheses in the posterior quadrant were excluded. A clinical dental evaluation excluded the presence of TMD symptoms. A clinical neurologic evaluation excluded the presence of neurologic swallowing disorders due to the central nervous system or to the peripheral cranial and cervical nerves. A clinical medical evaluation excluded the presence of salivary gland diseases. Subjects reported no difficulties in bolus (solid or liquid) swallowing during feeding.

The subjects were informed of the study procedure, but they ignored the study purpose. The Institutional Review Board approved the research with the understanding that the subjects would undergo electromyography and kinesiography for reasons independent of the research. Ethical approval was obtained by the University's Review Board for Health Sciences Research Involving Human Subjects and all subjects signed informed consent prior to testing.

### SEMG and kinesiographic measurements

The bilateral masseter, anterior temporalis, submental, and sternocleodomastoid muscles were recorded. Disposable silver/silver chloride bipolar surface electrodes (Duotrode; Myotronics-Noromed, Inc., Seattle WA, USA) with an interelectrode distance of 2.1 +/- 1.0 mm. and an impedance at 10.0 Hz max were used. Fifty ohms were placed on the bellies of the muscles parallel to the muscle fibers.

The same electrodes and cables were used for each patient.

The submental muscles were recorded to visualize activation of the muscle during swallowing.

The masseter and anterior temporalis muscles were recorded to show isometric activation during swallowing.

The sternocleidomastoid muscles were recorded to check the position of the head during swallowing and to evaluate isometric activity of the neck during swallowing. The myoelectric and kinesiographic signals were recorded using computerised equipment. This equipment allowed the recording of muscular activity and, at the same time, mandibular movement.

The SEMG was rectified and integrated. The SEMG data of each recorded muscle were expressed in microvolts.

Mandibular movement during swallowing were recorded by the kinesiograph of the K6-I Diagnostic System (Myotronics Research Inc., Seattle, WA, USA). The equipment consisted of an array of sensors placed on the subject's head that provided information about the position of the mandible. When the mandible moved, changes in the magnetic flux of the small bar magnet fixed on the mandibular incisor teeth were detected. The kinesiograph was connected to a computerized system that records and displays spatial coordinates in the vertical and anteroposterior axes to the nearest 0.1 mm.

**Figure 1:**
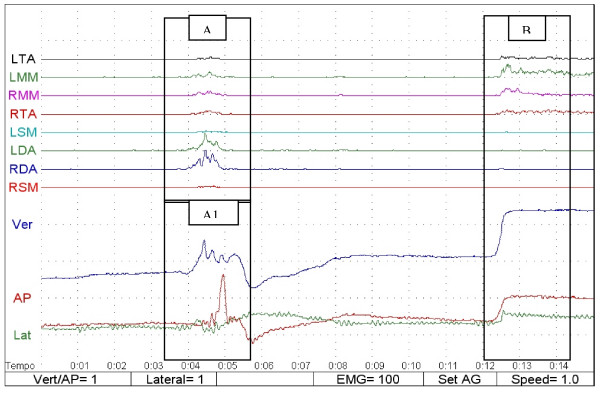
**Polygraphy of swallowing**: A = rectified SEMG during swallowing; A1 = kinesiography during swallowing, B = polygraphy in occlusal position; LTA (left anterior temporalis); RTA (right anterior temporalis); LMM (left masseter); RMM (right masseter); LSM (left sternocleidomastoid); RSM (right sternocleidomastoid); LDA (left submentalis); RDA (right submentalis). Ver (blue line): vertical component of mandibular movement; AP (red line): anteroposterior component of mandibular movement; Lat (green line): lateral component of mandibular movement.

When swallowing took place, the muscular pattern activation and mandibular movements were displayed on the video at the same time, allowing the observers to distinguish the swallowing function from other mandibular movements (Fig 1). The poligraphic gains used to display the SEMG and the kinesiographic values ere Vert/AP = 1 (each plotted square represented 1 mm of vertical or anteroposterior movement every second), Lateral = 1 (each plotted square represented 1 mm of lateral movement every second), EMG = 100 in processed mode (1 vertical division represented 100 microvolts of rectified SEMG and 1 horizontal division represented 1 second), set AG (represented the displayed muscles), and speed = 1 (represented the speed of recording 1 second for each horizontal division).

Kinesiograph was used in order to study the jaw position during spontaneous saliva swallowing. The distance between the deglutition and the occlusal position pointed out if swallowing was done in occlusal position or without occlusal contact.

**Figure 2:**
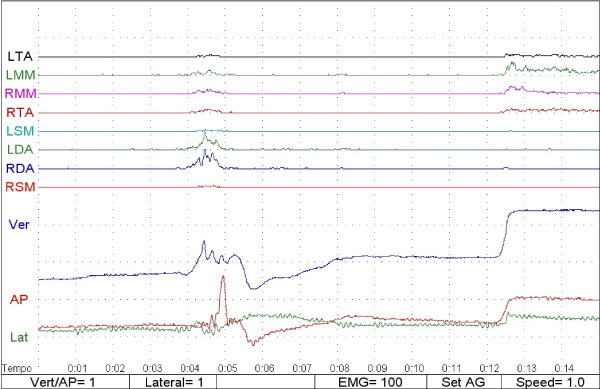
Swallowing without tongue interposition between dental arches (SNOC).

**Figure 3:**
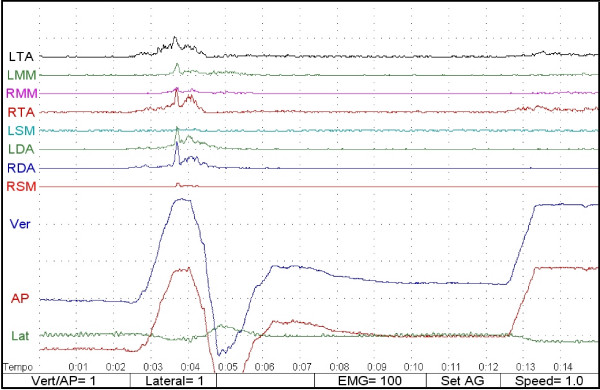
Swallowing with occlusal contact (SOC).

The different patterns of muscle activation and of mandibular movement allowed the observer to distinguish the swallowing event and to divide the sample into two groups: 1) swallowing with an occlusal contact (SOC) and 2) swallowing with no occlusal contact (SNOC; Figs. 2 and 3).

### Recording procedure

In order to assure spontaneous saliva swallowing the following recording protocol was used.

No information were given to subjects on study purpose. In other words study subjects didn't have any knowledge about electromyography, kinesiography and the reasons why they were tested. When the recording session started subjects had to close their eyes and wait, with closed eyes, for the end of recording session.

Polygraph recording session started after the electrodes placement and the magnet alignment.

Polygraph traces run on computer screen, when the traces reached the screen end, they were automatically deleted if the operator didn't recorder and save them. If the traces weren't recorded, they started to run the screen again. Operator could delete the traces at every time, in this way he could avoid that spontaneous saliva swallowing occurring at the end of the screen.

When a "spontaneous" saliva swallowing took place the operator waited for some seconds after the swallowing, and then asked the patient to reach the occlusal position. The operator recorded and saved the data of the first spontaneous saliva swallowing. After this first step, the computer was reset and it started again for another recording of spontaneous saliva swallowing. Three spontaneous saliva swallowing were recorded for each subject. Sometimes the procedure took fifteen or more minutes, in other cases it took five or less minutes, according to the variable frequency of swallowing in healthy people. The total duration of polygraph session wasn't recorded.

A "Controller" looked at the face of the subject during the whole recording session. This "controller" didn't have information about polygraph tracing, and he informed, lifting up one hand, on muscles and mandible movement occurring during swallowing. In this way study subject couldn't associate his swallowing with a voice signal.

When the third polygraph swallowing was recorded the study subject was asked to open the eyes and poligraph recording session was stopped.

### Measurement of the duration of swallowing

The polygraph was plotted on graph paper. A researcher trained in polygraph without knowledge of the study design measured the swallowing time using a SEMG submental activation. The time was computed from the start to the end of the submental muscle activation during the 3D movements on the kinesiographic trace. The measurement was done in seconds. For statistical analysis, an average of three measurements was used for each patient.

### Statistical analysis

A descriptive statistical analysis was done using the STATA package, both for the SEMG amplitude of each recorded muscle and for the duration of the swallowing.

A summary statistic was initially done on the entire sample.

Two comparison tests with independent samples (i.e., paired Student's t tests) were used to compare the mean value of the rectified SEMG and the mean duration time of swallowing with the SOC and SNOC groups. The level of significance was set at p < 0.05.

Analysis of variance (ANOVA) was performed to establish the influence of gender, age, and swallowing patterns (SOC-SNOC) on the SEMG values and on the duration of swallowing.

## Results

Table 1 shows the swallowing rectified SEMG (mean and sd) of the entire sample (111 patients). The submental muscles showed the highest SEMG activation (LDA, 25.42; RDA, 26.06). Masseter (LMM, 17.05; RMM, 16.30), and the anterior temporalis muscles (LTA, 17.81; RTA, 17.69) were activated during swallowing, but had a lower SEMG value than the submental group. The sternocleidomastoid muscles (LSC, 6.70; RSC, 6.91) reached the lowest activation values.

**Table 1:** Swallowing rectified SEMG of entire sample study (111 patients)

	LTA	LMM	RMM	RTA	LSC	LDA	RDA	RSC
Means	17.81	17.05	16.30	17.69	6.70	25.42	26.06	6.91
SD	19.17	17.09	14.59	20.84	5.25	12.43	11.83	4.39
SE	1.82	1.62	1.38	1.97	0.49	1.18	1.12	0.41

Values are represented in microvolts.

SD = standard deviation;

SE = standard error;

LTA = Left Temporalis Anterior;

LMM = Left Masseter;

RMM = Right Masseter;

RTA = Right Temporalis Anterior;

LSC = Left Sternoscleidomastoid;

LDA = Left Digastric;

RDA = Right Digastric;

RSC = Right Sternocleidomastoid

Table 2 shows the comparison test (i.e., paired Student's t tests) on the mean value of the rectified SEMG in the SOC and SNOC groups.

**Table 2:** SEMG swallowing values in SOC and SNOC group

	LTA	LMM	RMM	RTA	LSC	LDA	RDA	RSC
SOC	54	54	54	54	54	54	54	54

Means	26.24	24.09	22.94	27.16	6.38	25.59	26.87	6.83
SD	21.74	21.74	17.9	25.52	5.14	13.08	11.91	3.84
SE	3.18	2.95	2.43	3.47	0.69	1.78	1.62	0.52

SNOC	57	57	57	57	57	57	57	57

Means	9.82	10.38	10.01	8.71	7.0	25.26	25.29	7.0
SD	8.46	5.75	5.69	8.26	5.39	11.9	11.8	4.88
SE	1.10	0.76	0.75	1.09	0.71	1.57	1.56	0.64

p	0.0001	0.0001	0.0001	0.0001	0.2	0.4	0.2	0.3

Values are represented in microvolts

SOC = Subjects in Occlusal Contact;

SNOC = Subjects Not in Occlusal Contact;

SD = standard deviation; SE = standard error;

LTA = Left Temporalis Anterior;

LMM = Left Masseter;

RMM = Right Masseter;

RTA = Right Temporalis Anterior;

LSC = Left Sternoscleidomastoid;

LDA = Left Digastric;

RDA = Right Digastric;

RSC = Right Sternocleidomastoid

In agreement with the polygraphic analysis, 54 patients swallowed with occlusal contact and 57 patients swallowed without occlusal contact.

No significant differences existed in the SEMG activity of the submental muscles between the SOC and SNOC groups (LDA, p = 0.4; RDA, p = 0.2).

No significant differences existed in the sternocleidomastoid muscles between the SOC and SNOC groups (LSC, p = 0.2; RSC, p = 0.3).

A highly significant difference (p = 0.0001) existed between the SOC and SNOC groups in all the SEMG activation of the stomatognatic muscles (LTA, RTA, LMM, RMM).

Table 3 shows the comparison test (i.e., paired Student's test) on the mean duration of swallowing in the SOC and SNOC groups.

**Table 3:** Swallowing duration in SOC and SNOC groups

	SOC	SNOC
Swallowing duration (Means)	1.47	1.33 *
SD	0.48	0.38
SE	0.5	0.05

p		

Values are represented in seconds

SOC = Subjects in Occlusal Contact;

SNOC = Subjects Not in Occlusal Contact;

SD = standard deviation;

SE = standard error

* = p = 0.02

A significant difference (p = 0.02) existed in the duration of swallowing between the SOC (1.472 seconds) and SNOC (1.327 seconds) groups.

Tables 4, 5, 6 show the incidence on the muscles recorded by the SEMG of the swallowing patterns (SOC and SNOC), gender, and age.

**Table 4:** Analysis of variance (ANOVA): incidence of swallowing pattern on SEMG

Source	Partial SS	df	MS	F	Prob > F
LTA	7472.91	1	7472.91	24.69	0.0000
RTA	9436.58	1	9436.58	26.81	0.0000
LMM	5209.63	1	5209.63	21.09	0.0000
RMM	4633.77	1	4633.77	26.86	0.0000
LSC	10.35	1	10.35	0.37	0.5428
RSC	.77	1	.77	0.04	0.8426
LDA	3	1	3.	0.02	0.8898
RDA	68.54	1	68.54	0.49	0.4867

SS = Sum of Square

MS = Model of Sum of Square

F = Distribution of the Sample

**Table 5:** Analysis of variance (ANOVA): incidence of gender on SEMG

Source	Partial SS	df	MS	F	Prob > F
LTA	585.86	1	585.86	1.60	0.2084
RTA	1719.49	1	1719.49	4.07	0.0462
LMM	1105.25	1	1105.25	3.88	0.0513
RMM	725.59	1	725.59	3.48	0.0647
LSC	30.88	1	30.88	1.12	0.2925
RSC	41.94	1	41.94	2.20	0.1409
LDA	390.35	1	390.35	2.56	0.1126
RDA	394.94	1	394.94	2.87	0.0932

SS = Sum of Square

MS = Model of Sum of Square

F = Distribution of the Sample

**Table 6:** Analysis of variance (ANOVA): incidence of age on SEMG

Source	Partial SS	df	MS	F	Prob > F
LTA	8959.39	1	218.52	0.48	0.9938
RTA	14496.99	1	353.58	0.73	0.8577
LMM	9164.85	1	223.53	0.67	0.9146
RMM	6554.50	1	159.86	0.65	0.9284
LSC	898.64	1	21.92	0.71	0.8841
RSC	819.33	1	19.98	1.06	0.4083
LDA	8718.61	1	212.65	1.77	0.0182
RDA	5892.52	1	143.72	1.04	0.4312

SS = Sum of Square

MS = Model of Sum of Square

F = Distribution of the Sample

Gender and age did not explain the SEMG variability in all the muscles recorded.

The swallowing pattern (SOC and SNOC) accounted for the high statistical significance in the variability of the LTA, RTA, LMM, and RMM SEMG data, but was not able to account for the variability in the LSC, RSC, LDA, and RDA muscles.

Table 7 shows the incidence of gender, age, and swallowing pattern (SOC and SNOC) on the duration of swallowing.

**Table 7:** Analysis of variance (ANOVA): incidence of gender, age, and swallowing pattern (SOC, SNOC) on duration of swallowing.

Source	Partial SS	df	MS	F	Prob > F
sex	14.88	1	14.88	0.75	0.3898
age	904.81	41	22.07	1.18	0.2643
swpat	82.25	1	82.25	4.25	0.0416

SS = Sum of Square

MS = Model of Sum of Square

F = Distribution of the Sample

Swpat = Swallowing Pattern

Gender and age did not account for the variability in the duration of swallowing.

The swallowing pattern explained in a significant way (p = 0.04) the variability in the duration of swallowing.

## Discussion

In 1953, Jankelson [26] stated that the only physiologic occlusal contact during mastication occurs when swallowing takes place. Occlusal stability is needed to give a skeletal support to the muscular events related to swallowing [27]. Up to now, some orthodontic schools aim to obtain correct swallowing when the occlusal contact is reached during swallowing.

Nevertheless, recent findings [19,24,25] have suggested that the interposition of the tongue between the dental arches during swallowing is a physiologic event.

Our data confirmed the data of Monaco [24] on spontaneous saliva swallowing and Sadalla [19] on voluntarily dry swallowing regarding the incidence of swallowing without an occlusal contact.

In our sample of healthy patients, 57 of 111 swallowed without any occlusal contact and 54 had occlusal contact. Sadalla, in a kinesiographic study, stated that 70% of dentate patients swallowed with the interposition of the tongue between the teeth arches.

According to Erkin et al. [15] who suggested a different nervous control and peripheral muscular pattern of spontaneous and voluntarily swallows, the difference reported in Monaco and Sadalla studies could be due to different method and different swallow protocol used. Furthermore, Monaco studied the spontaneous saliva svallowing, Sadalla investigated the voluntarily dry swallowing. Nevertheless, swallowing without occlusal contact seems to be frequent in healthy people.

Monaco, moreover, suggested that, swallowing with the tongue interposition could be considered a normal pattern of muscular activation during spontaneous saliva swallowing. Monaco dividing the study sample into sub-diagnosis groups, pointed out that spontaneous saliva swallowing with an occlusal contact is more frequent in people who received a prosthetic treatment [24]. She noticed that the neuromuscular pattern requiring occlusal contact during deglutition could be unfavourable for the health of the stomatognathic system if some unbalanced muscular forces developed in the occlusion.

Logeman [28] stated that spontaneous saliva swallowing contain about 1 ml of saliva; it's very likely that such small amount of liquid could be swallowed by either occlusal or no-occlusal contact in healthy people.

The fact that a small amount of bolus (i.e. 1 ml saliva) can easily be swallowed by either occlusal or no occlusal contact doesn't means that a preferred spontaneous pattern of swallowing doesn't exist.

Dental practice and Orthodontics are facing, daily, on the muscular forces exerted by tongue, lips, cheeks, masseter, temporalis anterior etc. during spontaneous saliva swallowing on teeth and on neck muscles.

One of the signs of "Atypical swallowing" is the interposition between dental arches of the tongue. The effect of the interposition is the no-occlusal contact between the dental arches. This spontaneous swallowing pattern is considered one of the causes of malocclusion.

Our study suggests that in healthy people without objective or subjective swallowing disorders there are two different preferred polygraph patterns of spontaneous swallowing.

A previous kinesiographic work [24] suggested that spontaneous saliva swallowing without occlusal contact it isn't more frequently associated than spontaneous saliva swallowing with occlusal contact to Temporo Mandibular Disorder (TMD) or to teeth loss and prosthetic treatment. On the contrary, spontaneous saliva swallowing with occlusal contact seemed to be more frequently related to teeth loss and prosthetic treatment than spontaneous saliva swallowing without occlusal contact.

Monaco conclusions were based on kinesiographic study and couldn't relate muscular activation or tip of tongue position during mandible movement.

Despite of the fact that dental practitioners assign considerable importance to the physiology of swallowing, [1,2,26,27], few works have studied the stomatognathic muscular pattern of this event [22,23].

The rectified SEMG of the submental muscles has been recently used to study the physiology of swallowing. The SEMG appears to be a reliable, simple, and noninvasive tool to analyse the physiologic events related to swallowing. In particular, the SEMG of the submental muscles seems to be related to the elevation of the hyoid bone and its return to a resting position.

In our study, the SOC and SNOC groups showed the same amplitude in the SEMG of the submental muscles, suggesting that the forces released by these muscles, despite the spontaneous saliva swallowing pattern, were comparable.

In agreement with other authors [6,7,9,11] the submental SEMG allows screening of some normal and non-physiologic characteristics of swallowing. Our study group was comprised of healthy subjects without TMD or swallowing disorders, therefore, the submental SEMG showed the physiologic activation of the muscles. The submental SEMG gave little information about the forces released on the neck and on the stomatognathic structures by muscular activation during swallowing.

The sternocleidomastoid muscles showed a slight, but constant activation during spontaneous saliva swallowing. The neck muscles contributed to stabilizing the head on the trunk during the isometric work done by the mandibular elevator muscles [29]. Our data suggest that the same activity takes place during spontaneous saliva swallowing and that the sternocleidomastoid muscles of the SOC and SNOC groups activated themselves in the same way, discharging on the neck and on the trunk the forces exerted during spontaneous saliva swallowing. This finding is noteworthy for the influence on the body posture exerted by the physiologic stomatognathic acts.

Our data confirm that during deglutition, the masseter and anterior temporalis muscles activate themselves at the same time with the submental and sternocleidomastoid muscles.

The rectified SEMG showed an increase of electrical potential from these muscles.

The rectified SEMG increased when the isometric contraction took place. An increase in the electrical potential during swallowing goes along with the mandibular stabilization obtained with the isometric contraction of the recorded muscles.

During isometric contraction, the increase of the myoelectrical potential is related in a non-linear way to the increase in the force generated by the muscles [16,17].

During spontaneous saliva swallowing this force is projected on the teeth and on the skeletal structure of the face, head, and neck.

The frequency [3,12] of swallowing during the day and night justifies the molding action of this function on the skeletal structure.

In our sample the SOC and SNOC groups behaved in a different way.

The activity of the masseter and the anterior temporalis muscles in the SOC group was significantly higher than in the SNOC group. The data in the present study confirmed the findings of Wilson [3] and Moriniere [12], who stated that the duration of swallowing in healthy adult people ranges from 0.80 to 1.60 seconds. In our sample, the duration range of swallowing in the SOC group was significantly higher than in the SNOC group, but the swallowing duration of both groups is included in 0.8–1.6 range. It is possible that this range consider spontaneous saliva and voluntarily dry swallowing.

Ertekin [15] suggested that spontaneous saliva swallowing lasted less than 1 sec., and asserted that swallowing lasting more than 1 sec. could be driven by some kind of voluntarily action.

Swallowing duration in our sample lasted 1.327 sec. in NSOC and 1.472 in SOC. We can't exclude that some voluntarily drive affected our data, but such drive would be randomly distributed over the entire sample, and it wouldn't compromise the significant difference of swallowing duration between the SOC and SNOC group, and it wouldn't invalidate the general significance of the results for dental practice and orthodontics in healthy individuals.

Moreover, gender and age were not related to the activation of the SEMG during swallowing, while the swallowing pattern was related to the SEMG and to the duration of swallowing. People who swallow with an occlusal contact had higher SEMG values of the masseter and anterior temporalis muscles and higher values for the duration of swallowing.

These data suggest that during swallowing with an occlusal contact, higher and more prolonged forces are released on the teeth and on the skeletal structure partly justifying Monaco's findings. [24] It is possible that people with a swallowing neuromuscular pattern of the SOC type and with an occlusal plane in disharmony with the muscular forces, have suffering involving the teeth and periodontium. In contrast, people with a swallowing neuromuscular pattern of SNOC type could use the tongue like a bite and could counterbalance a possible disharmony between the occlusal plane and the muscular forces. In this case, the teeth could change position, but would not suffer from a periodontal loss.

Further work is needed to support these considerations; in particular, it would be interesting to compare the polygraph pattern of spontaneous saliva swallowing and voluntarily dry swallowing in order to investigate the difference of this two kind of deglutition, and to analyze the symmetry of activation of the muscles during swallowing in order to detect the occlusal disharmony and to relate it with periodontal diseases or loss of teeth.

## Conclusion

The polygraph seems to be a valuable, simple, non-invasive, and reliable tool to study the physiology of swallowing.

Even if SOC and SNOC behave in different ways, our data suggest that there is not a single normal or typical pattern for swallowing in healthy subjects without objective or subjective signs or symptoms of swallowing disorders. It is possible to suggest that the interposition of the tongue between the dental arches during swallowing could be a physiologic event similar to swallowing with an occlusal contact.

No significant differences existed in the SEMG activity of the submental muscles between the SOC and SNOC groups.

No significant differences existed in the sternocleidomastoid muscles between the SOC and SNOC groups.

A highly significant difference (p = 0.0001) existed between the SOC and SNOC groups in all the SEMG activation of the stomatognatic muscles (LTA; RTA, LMM, RMM).

This work allows describing polygraph of muscle activity and mandible movement during spontaneous saliva swallowing in healthy subjects, showing normative data for dental use.

Furthermore, this study could be clinically relevant suggesting objective data (swallowing duration and muscle activity pattern) in order to check dental therapy (prosthetic and orthodontic treatment).

## Competing interests

The author(s) declare that they have no competing interests.

## Authors' contributions

MA provided the study design, the coordination of the data collection, the manuscript conception, the drafting, and review of the article. CR reviewed the data collection, provided the analysis and the interpretation of the data, and contributed to the manuscript conception. SA participated in the collection and interpretation of the data and contributed to the drafting of the manuscript. GM participated in the study design and significantly contributed to the review of the manuscript. All authors read and approved the final manuscript.

## Pre-publication history

The pre-publication history for this paper can be accessed here:


